# Exploring Lead loci shared between schizophrenia and Cardiometabolic traits

**DOI:** 10.1186/s12864-022-08766-4

**Published:** 2022-08-25

**Authors:** Qian He, Adam N. Bennett, Jundong Liu, Beifang Fan, Xue Han, Lu Cheng, Yan Chen, Xia Yang, Kei Hang Katie Chan

**Affiliations:** 1 Department of Biomedical Sciences, City University of Hong Kong, Hong Kong, SAR China; 2 Department of Mental Health, Shenzhen Nanshan Center for Chronic Disease Control, ShenZhen, China; 3 Department of Integrative Biology & Physiology, University of California, Los Angeles, USA; 4 Brain Research Institute, University of California, Los Angeles, USA; 5 Institute for Quantitative and Computational Biosciences, University of California, Los Angeles, USA; 6 Department of Electrical Engineering, City University of Hong Kong, Hong Kong, SAR China; 7 Department of Epidemiology, Centre for Global Cardiometabolic Health, Brown University, Providence, RI USA

**Keywords:** Schizophrenia, Cardiometabolic traits, Conditional FDR, Conjunctional FDR, Susceptibility gene

## Abstract

Individuals with schizophrenia (SCZ) have, on average, a 10- to 20-year shorter expected life span than the rest of the population, primarily due to cardiovascular disease comorbidity. Genome-wide association studies (GWAS) have previously been used to separately identify common variants in SCZ and cardiometabolic traits. However, genetic variants jointly influencing both traits remain to be fully characterised. To assess overlaps (if any) between the genetic architecture of SCZ and cardiometabolic traits, we used conditional false discovery rate (FDR) and local genetic correlation statistical framework analyses. A conjunctional FDR was used to identify shared genetic traits between SCZ and cardiometabolic risk factors. We identified 144 genetic variants which were shared between SCZ and body mass index (BMI), and 15 variants shared between SCZ and triglycerides (TG). Furthermore, we discovered four novel single nucleotide polymorphisms (SNPs) (rs3865350, rs9860913, rs13307 and rs9614186) and four proximate genes (*DERL2*, *SNX4*, *LY75* and *EFCAB6*) which were shared by SCZ and BMI. We observed that the novel genetic variant rs13307 and the most proximate gene *LY75* exerted potential effects on SCZ and BMI comorbidity. Also, we observed a mixture of concordant and opposite direction associations with shared genetic variants. We demonstrated a moderate to high genetic overlap between SCZ and cardiometabolic traits associated with a pattern of bidirectional associations. Our data suggested a complex interplay between metabolism-related gene pathways in SCZ pathophysiology.

**Supplementary Information:**

The online version contains supplementary material available at 10.1186/s12864-022-08766-4.

## Background

Individuals with schizophrenia (SCZ) have a 10- to 20-year shorter life span when compared with healthy individuals in the same population [1, 2]. Previous studies have indicated that cardiovascular disease could be a major cause of this shorter life expectancy in SCZ patients [1]. The link between the increased incidence of cardiovascular and metabolic disorder was previously established in SCZ patients when compared with the general population [2, 3]. For example, the risk of obesity and type 2 diabetes (T2D) are approximately 3.5- and 2-fold higher, respectively, in individuals with SCZ [4, 5]. Historically, the increased risk and prevalence of cardiometabolic disease (CMD) has been attributed to social determinants and lifestyle factors (including poor diet, sedentary behaviour and alcohol and substance use) and the effects of psychotropic medication [6, 7]. Furthermore, several psychopharmacological agents, in particular antipsychotics, are obesogenic and contribute to adverse events due to metabolic disorders [7] These factors suggest that CMD risks are both key risk factors and long-term health concerns in patients with SCZ [8]. However, for decades, cardiometabolic comorbidity and associated mortality have remained high in these patients and have suggested that most patients with SCZ have not benefited from clinical advancements [8, 9]. Therefore, comorbidity may be a result of other factors hitherto not considered. Therefore, a systems biology approach could provide new pathophysiological knowledge as indicated by recent genetics studies [10, 11].

Recently, the polygenic nature of SCZ and cardiometabolic traits have become increasingly clear [12]. These traits are reported with substantial heritability, estimated at 79% for SCZ [13], 24–90% for BMI [14], 36–61% for waist-hip ratio (WHR) [15], 11% for triglycerides (TG) [16], approximately 89% for total cholesterol (TC), 22–93% for high-density lipoprotein (HDL), 22–91% for low-density lipoprotein (LDL), 38–66% for fasting glucose (FG) [17], 47% for fasting insulin (FIN) [18] and 25–80% for T2D [19]. Several genetic studies have established links between CMD and SCZ, and an increased CMD prevalence has been associated with treatment responses in SCZ [20, 21]. Due to strong associations between BMI and SCZ, and also between TG and SCZ [22, 23], several neurobiological hypotheses related to potential underlying mechanisms have been proposed. However, associations are complex, as weight loss [24, 25] and weight gain [26] are associated with SCZ, and inconsistent associations between TG and SCZ have been reported [27, 28].

Even though abundant genetic variants are associated with SCZ comorbidity and cardiometabolic traits, understanding the functional consequences of genetic variations and identifying pleiotropic genes and pathways for both phenotypes remains challenging. Powerful statistical approaches, specifically designed to analyse the polygenic architectures of complex traits, could improve gene or loci discovery and replication rates [25–27]. The conditional FDR and conjunctional FDR methods, which specifically analyse the polygenic architecture of multiple disorders, allow for the identification of shared genetic variants, and in turn, elucidate common pathobiology and molecular mechanisms across different disorders [10]. Using this strategy, common associations between two phenotypes can be identified by evaluating the contribution from all SNPs from two independent GWAS [28–30]. The discovery of shared genetic variants could facilitate the development of risk prediction models for CMD traits and enabling targeted CMD interventions for SCZ patients.

In this study, we analysed GWAS summary statistics of SCZ and cardiometabolic traits, including WHR, BMI, TG, TC, HDL, LDL, FG, FIN and T2D, using pleiotropic-based conditional and conjunctional FDR statistics to estimate shared genetic characteristics between SCZ and cardiometabolic traits. We hypothesised that these methods could help identify shared genetic variants, shared polygenic architecture, and potential pleiotropic genes and biological pathways shared between SCZ and cardiometabolic traits.

## Results

### Polygenetic overlap and genetic correlations between SCZ and Cardiometabolic traits

**Fig. 1:**
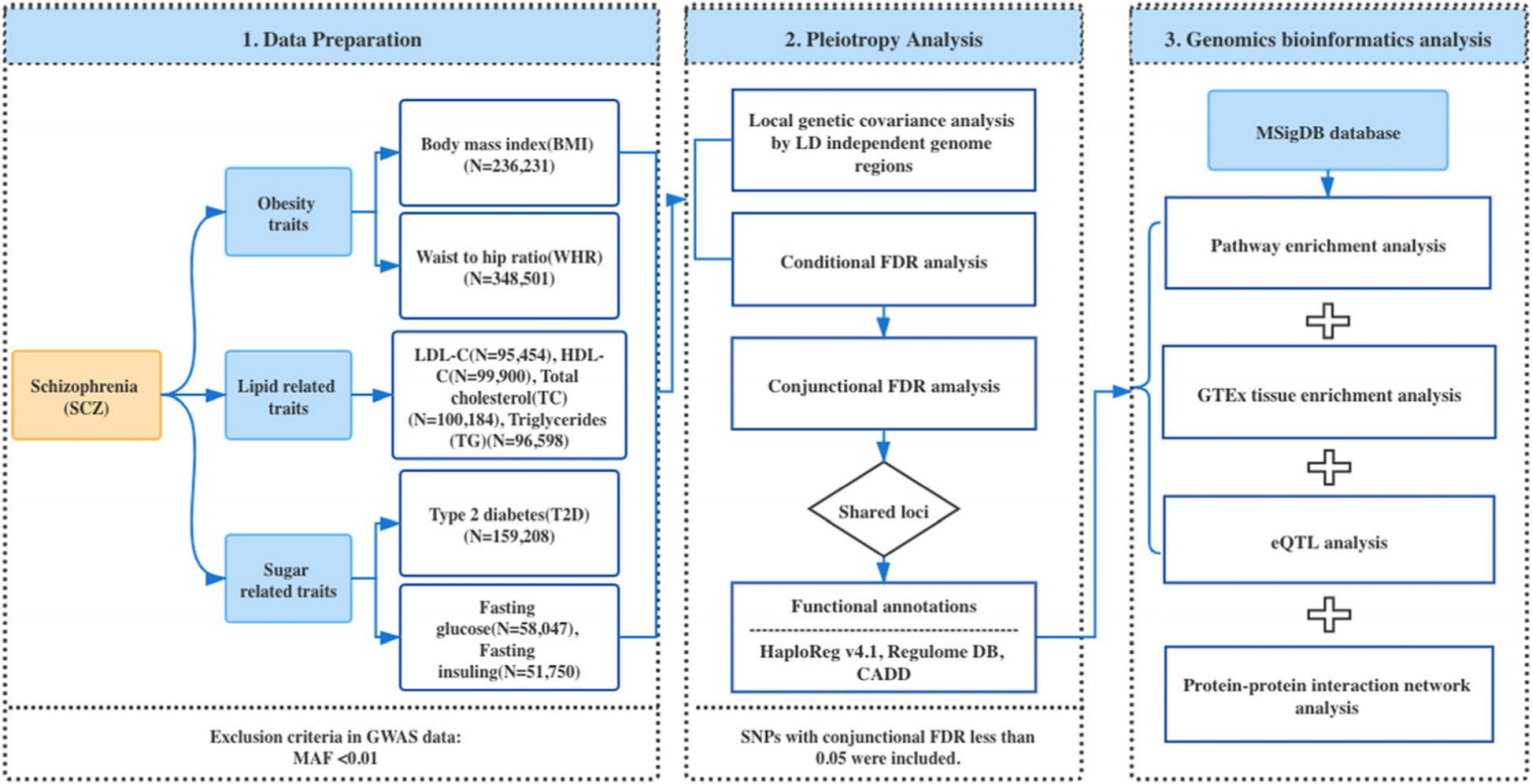
Work flow of the study design

As shown (Fig. 1), we performed conditional FDR and local genetic covariance analyses to identify pleiotropic effects between SCZ and cardiometabolic traits in a European ancestry background. Briefly, the conditional FDR approach was based on an empirical Bayesian statistical framework and used GWAS summary statistics as a primary trait (e.g. SCZ), together with a conditional trait (e.g. BMI) to estimate the posterior probability that an SNP had no association with the primary trait, given that *P*-values for that SNP in both primary and conditional traits were as small as, or smaller than the observed *P*-value.

**Fig. 2:**
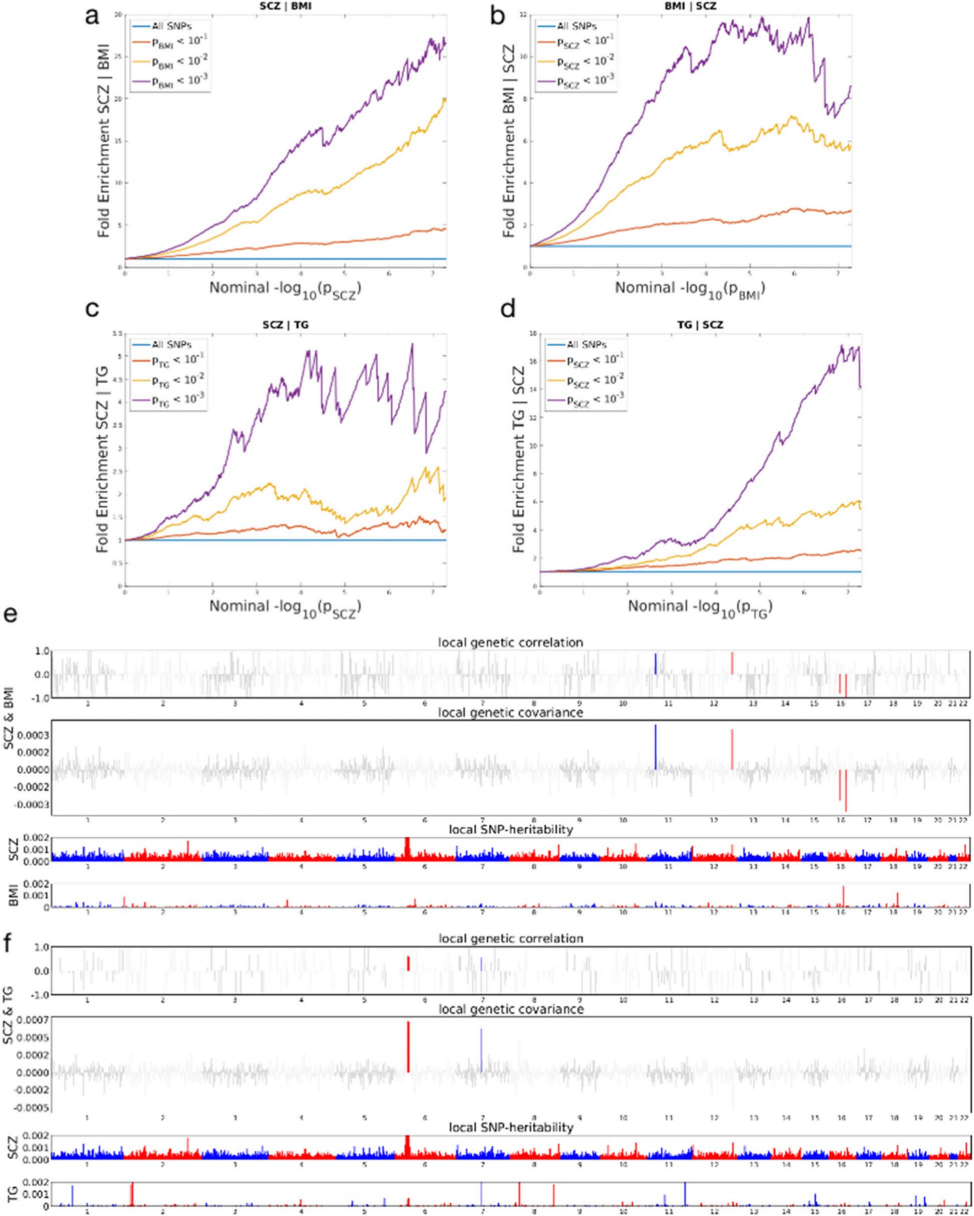
Pleiotropy analysis. **a** Plot of fold enrichment vs. nominal -log10 *P* for SCZ below the standard GWAS threshold of *P* value < 5 × 10^− 8^ as a function significant of the association with BMI. **b** Plot of fold enrichment versus nominal -log10 *P* values for BMI below the standard GWAS threshold of *P* value < 5 × 10^− 8^ as a function of significance of the association with SCZ. **c** Plot of fold enrichment vs. nominal -log10 *P* for SCZ below the standard GWAS threshold of *P* value < 5 × 10^− 8^ as a function of significance of the association with TG. **d** Plot of fold enrichment of TG versus nominal -log10 *P* values for below the standard GWAS threshold of *P* value < 5 × 10^− 8^ as a function of significance of the association with SCZ. **e** Local genetic correlation and local SNP-heritability between SCZ and BMI. **f** Local genetic correlation and local SNP-heritability between SCZ and TG. For each panel (e&f), the top section represents local genetic correlation, the middle section represents local genetic covariance, where significant local genetic correlation and covariance after multiple testing correction are highlighted in blue; and the bottom part represents local SNP-heritability for individual trait

Our fold-enrichment plots demonstrated that SNPs were highly enriched (4.8–25.0 fold) in SCZ, across increasingly stringent significance levels, for a BMI association (SCZ|BMI) (Fig. 2a), while SNPs were moderately enriched (1.3–4.3 fold) in SCZ, across increasing stringent significance levels, for the SCZ|TG association (Fig. 2c). The reverse conditional association (BMI|SCZ and TG|SCZ) showed a ~ 2.5–9.0 fold and ~ 2.5–34.0 fold-enrichment, respectively (Fig. 2b, d). These results supported a moderate to high level of polygenic overlap between SCZ and BMI or TG.

We also used the Heritability Estimation from Summary Statistics (HESS) package to estimate and visualise local SNP-heritability and genetic covariance, to examine if a specific genomic region was genetically linked to SCZ and cardiometabolic traits. We estimated local genetic covariance and correlations in 104 regions between SCZ and BMI, and 32 regions between SCZ and TG. In analysis between two trait pairs (SCZ|BMI and SCZ|TG), we identified four genomic regions on different chromosomes: chr11:27020461–28,481,593, chr12:122007651–124,977,980, chr16:29036613–31,382,943 and chr16:63691589–65,938,566, which showed strong local genetic associations between SCZ and BMI (Fig. 2e and Additional file 1 Supplementary Table 2). We identified two genomic regions: chr6:31571218–32,682,664 and chr7:71874885–73,334,602 which showed strong local genetic correlations between SCZ and TG (Fig. 2f and Additional file 1 Supplementary Table 3).

### Genetic variants and genes identified by conjunctional FDR analysis are shared between SCZ and BMI and TG

**Fig. 3:**
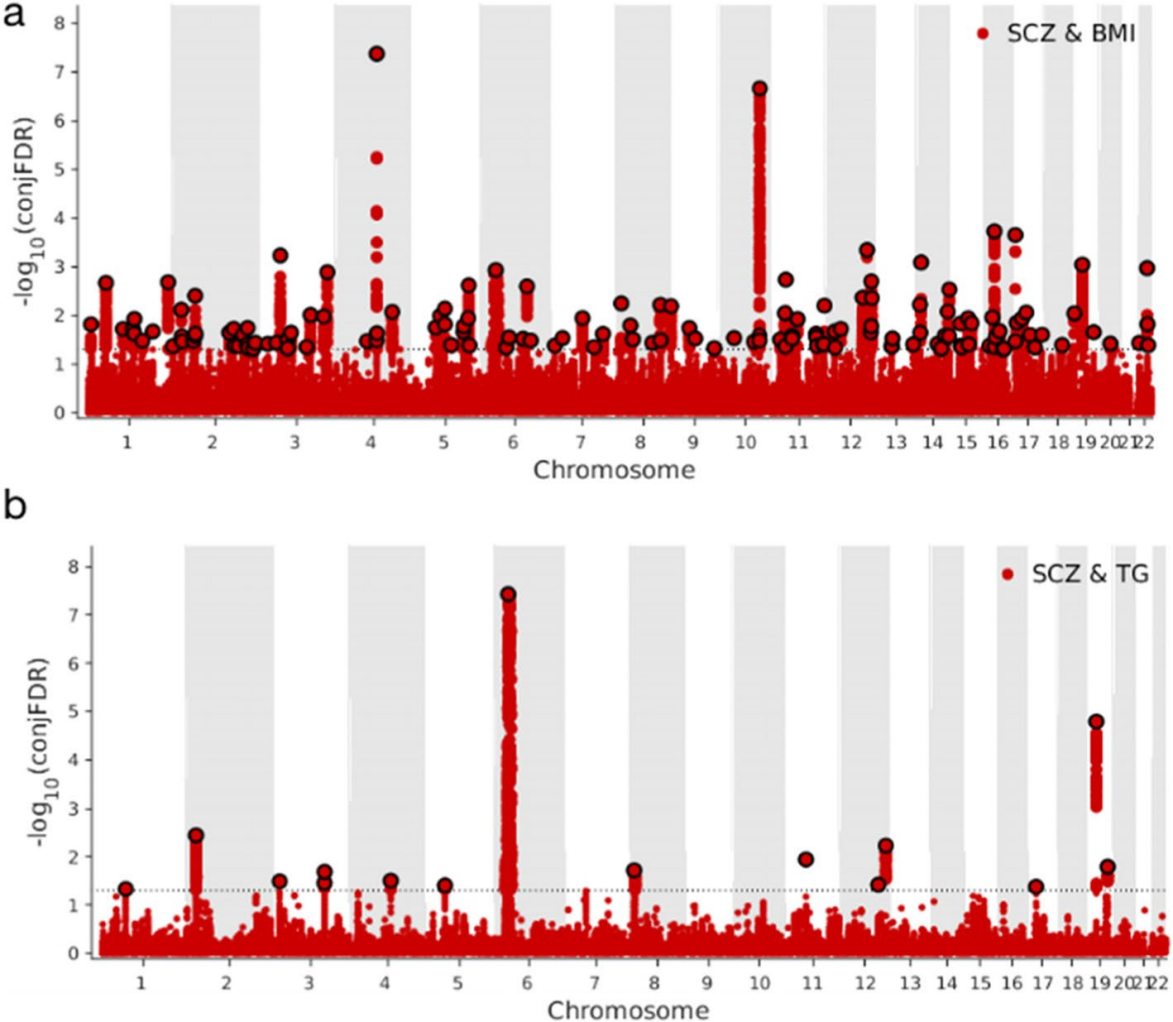
Common genetic variants jointly associated with SCZ and BMI (3a) and TG (3b) at conjunctional false discovery rate (conjFDR) less than 0.05. Manhattan plot showing the –log10 transformed conjFDR values for each SNP on the y-axis and chromosomal position along the x-axis. The dotted horizontal line represents the threshold chosen for reporting shared associations (−log (FDR) values of 1.3 corresponds to a cFDR ≤0.05). Independent lead single-nucleotide polymorphisms are highlighted with a black outline. The significant shared signal in the major histocompatibility complex region (chr6:25119106–33,854,733 and chr8:7242715–12,483,982) were deleted in the analysis. Further details are provided in Additional file 1 Supplementary Table 4 and Supplementary table 4_1

To identify genetic variants shared between SCZ and BMI and TG, we performed conjunctional FDR analysis. This approach assessed the posterior probability that an SNP was null for either trait or both, given that *P*-values for both phenotypes were as small as, or smaller than *P*-values for each trait individually. Conjunctional FDR is an extension of the conditional FDR approach and is defined as the maximum of two conditional FDR statistics for a specific SNP. In total, 144 distinct genetic variants were shared between SCZ and BMI at a conjunctional FDR value < 0.05 (Fig. 3a and Additional file 1 Supplementary Table 4). Of these, 80 variants (56%) were not found in the original BMI GWAS [31], while 75 (52%) were not found in the original SCZ GWAS [32] and 79 (55%) were not reported in similar studies [3, 31]. Four genetic variants were identified which were novel for both phenotypes and, after mapping the most proximate genes to associated SNPs, we identified *DERL2*, *SNX4*, *LY75* and *EFCAB6* as novel genes in SCZ and BMI associations (Table 1). By integrating information from local genetic covariance analyses, we identified six SNPs [rs6265 (chr11:27679916), rs7975482 (chr12:124481690), rs4243232 (chr16:30514723), rs7953704 (chr12:122625992), rs10744211 (chr12:122931820) and rs4787491 (chr16:30015337)] and six most proximate genes *BDNF*, *ZNF664*, *ITGAL*, *MLXIP*, *ZCCHC8* and *INO80E* at three significant genomic regions (chr11:27020461–28,481,593, chr12:122007651–124,977,980 and chr16:29036613–31,382,943). All genes were previously reported as being associated with BMI and SCZ.

**Table 1:** Novel shared genes reaching statistical significance between SCZ and cardiovascular risk traits (BMI and TG) (conjFDR ≤ 0.05)

SNP	A1	A2	CHR	BP	conjFDR	Function	ANNOVAR	RDB	CADD	minChrState	commonChrState	Beta_BMI	SE_BMI	P_BMI	OR_SCZ	SE_SCZ	P_SCZ
Novel SNPs and Genes shared between SCZ and BMI																	
rs3865350	C	T	17	5,381,867	0.01.45	intronic	*DERL2*	6	0.393	4	4	-0.0142	0.0036	8.00 × 10^-5^	1.0414	0.0097251	2.97 × 10^-5^
rs9860913	A	G	3	125,137,326	0.04.48	intergenic	*SNX4*	5	3.826	5	15	0.0181	0.006	2.56 × 10^-^^3^	0.93514	0.016112	3.15 × 10^-5^
rs13307	G	A	2	160,659,996	0.02.28	UTR3	*LY75*	4	3.736	5	15	0.0129	0.0036	3.39 × 10^-4^	1.0393	0.009675	6.75 × 10^-5^
rs9614186	A	C	22	44,187,687	0.041	intronic	*EFCAB6*	4	4.526	4	15	0.0147	0.0041	3.12 × 10^-4^	1.0516	0.013542	2.02 × 10^-4^
Novel SNPs and Genes shared between SCZ and TG																	
SNP	A1	A2	CHR	BP	conjFDR	Function	Gene	RDB	CADD			Beta_TG	SE_TG	P_TG	OR_SCZ	SE_SCZ	P_SCZ
rs1472584	G	A	5	45,210,282	3.95 × 10−-2	intergenic	*HCN1*	7	13.21	5	15	0.0218	0.0062	5.234 × 10^-4^	0.9491	0.012559	3.18 × 10^-5^
rs3130544	C	A	6	31,058,340	3.79 × 10−-8	intergenic	*C6orf15*	3a	2.903	NA	NA	-0.043	0.0075	4.84 × 10^-10^	1.1867	0.015159	1.42 × 10^-^^29^

Notes: *A1* Effect allele, *A2* Alter allele, *CHR* Chromosome number, *BP* Base-pair position, *conjFDR* Conjuctional false discovery rate, *ANNOVAR* Functional variant classification based on position in or outside of a gene, *RBD* RegulomeDB scores predicts the likelihood of regulatory functionality (lower scores, less than 3, indicate a higher likelihood), *CADD* Combined Annotation-Dependent depletion score, which predicts how deleterious the SNP effect is on protein structure/function (higher scores indicate more deleterious), *minChrState* Minimum chromatin state across 127 tissue types (lower scores indicate more open chromatin), *commonChrState* Most common chromatin state in 127 tissue types

In total, 15 genetic variants were shared between SCZ and TG at a conjunctional FDR value < 0.05 (Fig. 3b and Additional file 1 Supplementary Table 4_1). Of these, 10 (67%) genetic variants were not previously reported in the original TG GWAS [33]; while 10 (67%) were not previously reported in the original SCZ GWAS, and 6 (40%) were not previously reported in a similar study [3]. One genetic variant rs1472584, when the most proximate gene was mapped to the associated SNP, defined *HCN1* as a novel gene for TG, and was associated with SCZ in a previous study (Table 1 and Additional file 1 Supplementary Table 4_1). When we integrated information from local genetic covariance analysis, we identified one SNP rs3130544 and one proximate gene *C6orf15* in a significant genomic region (chr6:31571218–32,682,664). This novel TG gene was associated with SCZ (Table 1). By comparing the association direction for the top SNPs shared between SCZ and BMI at a conjunctional FDR value < 0.05, we identified mixed association direction patterns, with SNPs having concordant association directions in 72/144 genetic variants (50.0%) shared between BMI and SCZ (Additional file 1 Supplementary Table 5–6), while 8/15 genetic variants (53.3%) had concordant association directions between TG and SCZ (Additional file 1 Supplementary Table 7–8).

### Annotating genetic variants shared between SCZ and BMI and TG

The functional annotation of SNPs at a conjunctional FDR value < 0.05 for SCZ and BMI is shown (Additional file 1 Supplementary Table 4 and Additional file 2 Supplementary Fig. 1). Most SNPs were in intronic (54.29%) and intergenic (31.43%) regions, and 12.77% had a RegulomeDB score < 3, predicting potential regulatory functions (Additional file 2 Supplementary Fig. 1a). Details on RegulomeDB scores are shown (Additional file 1 Supplementary Table 9).

After functional annotation using ANNOVAR, we identified four novel SNPs (rs3865350, rs9860913, rs13307 and rs9614186) and four most proximate genes (*DERL2*, *SNX4*, *LY75* and *EFCAB6*) shared between SCZ and BMI. Ten candidate SNPs, in strong linkage disequilibrium (LD) (*r*^*2*^ ≥ 0.8) with rs3865350 (*DERL2*) at 17p13.2, were extracted using the HaploReg v4.2 tool (Additional file 1 Supplementary Table 10). We observed that rs3865350 was predicted to alter the binding of two transcription factor (TF) motifs GCNF and PLZF in HaploReg, which was not confirmed in GVATdb. In total, 42 candidate SNPs in strong LD (*r*^*2*^ ≥ 0.8) with rs9860913 (*SNX4*) at 3q21.2 were identified (Additional file 1 Supplementary Table 11). Using HaploReg, this locus was located within an activated enhancer and DNAse site in different cell types, but was not confirmed in EnhancerDB. We also identified 67 candidate SNPs in strong LD (*r*^*2*^ ≥ 0.8) with rs13307 (*LY75*) at 2q24.2 (Additional file 1 Supplementary Table 12). The binding site of three TF motifs (Evi-1, RPEB-1 and RXR) were affected by this variant in HaploReg, which was confirmed in GVATdb. In total, eight candidate SNPs in strong LD with rs9614186 (*EFCAB6*) at 22q13.2 were identified (Additional file 1 Supplementary Table 13).

The functional annotation of SNPs at conjunctional FDR < 0.05 for SCZ and TG are shown (Additional file 2 Supplementary Fig. 1). Most SNPs were within intronic (35.71%) or intergenic (42.86%) regions, and 6.67% had a RegulomeDB score < 3 (Additional file 2 Supplementary Fig. 1b).

We identified two novel SNPs (rs1472584 and rs3130544) and two most proximate genes (*HCN1* and *C6orf15*) which were not previously associated with TG, but with SCZ. Ninety candidate SNPs, in strong LD (*r*^*2*^ ≥ 0.8) with rs1472584 (*HCN1*) at 5p12, were extracted (Additional file 1 Supplementary Table 14). Additionally, the binding site of the TF motif, Sox, was affected by this variant in HaploReg, but not confirmed in GVATdb. Thirteen candidate SNPs, in strong LD (*r*^*2*^ ≥ 0.8) with rs3130544 (*C6orf15*) at 6p21.33, were extracted (Additional file 1 Supplementary Table 15). The binding site of the TF motifs CEBPα, ERα-a and RORα1 were affected by this variant in HaploReg, but this was not confirmed in GVATdb.

### Pathway analysis of genetic variants shared between SCZ and BMI and TG

**Fig. 4:**
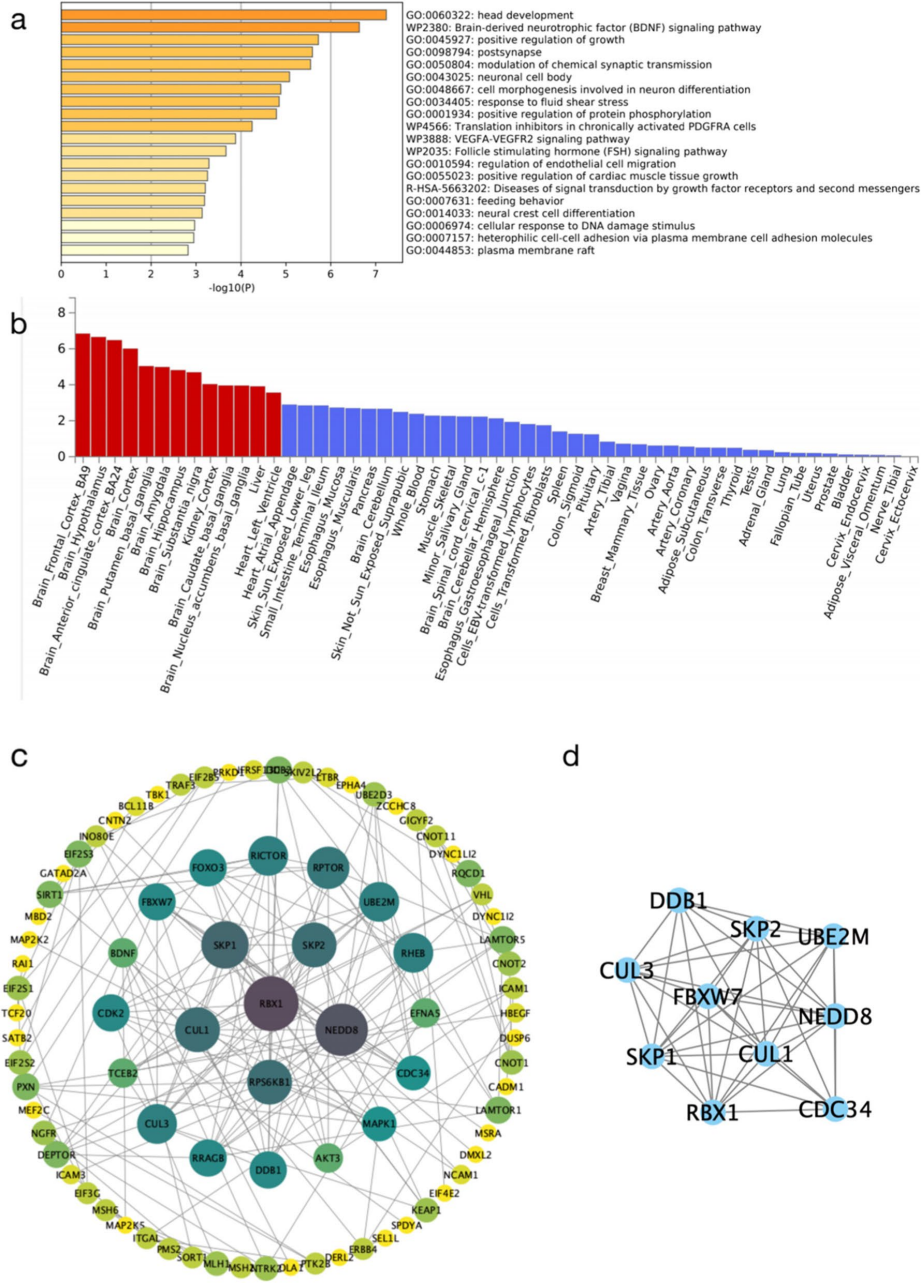
Functional enrichment analysis of shared genes between SCZ and BMI. a Pathway enrichment analysis. b Tissue enrichment analysis using 53 tissues from the GTEx database (version 7). Significantly enriched differential expressed gene (DEG) sets (Bonferroni corrected *P* < 0.05) are highlighted in red. c PPI network of the shared genes between SCZ and BMI. d Significant cluster related to the PPI network (Module1)

We performed pathway enrichment analyses for shared genetic variants between SCZ and BMI, and SCZ and TG, to separately identify overrepresented pathways among most proximate genes nearest identified genetic variants. For SCZ and BMI, 297 pathways were significantly overrepresented and related to central nervous system (CNS) neuron differentiation, brain-derived neurotrophic factor (BDNF) signalling, positive regulation of growth, post synapse and modulation of chemical synaptic transmission (Fig. 4a and Additional file 1 Supplementary Table 16). Gene analyses were consistent and opposite association directions were separately identified between BMI and SCZ and indicated only a minor overlap in overrepresented pathways. Concordant genes were enriched in heterocycle catabolic processes, BDNF signalling, neuronal cell bodies, the PIP3 activation of AKT signalling, intracellular signalling by second messengers and stem cell differentiation. Opposite genes were enriched in cellular responses to fluid shear stress, CNS neuro differentiation, excitatory postsynaptic potential, chemical synaptic transmission, postsynaptic transmission and the regulation of postsynaptic membrane potential. More results from genetic analyses are shown (Additional file 1 Supplementary Tables 17 and 18).

**Fig. 5:**
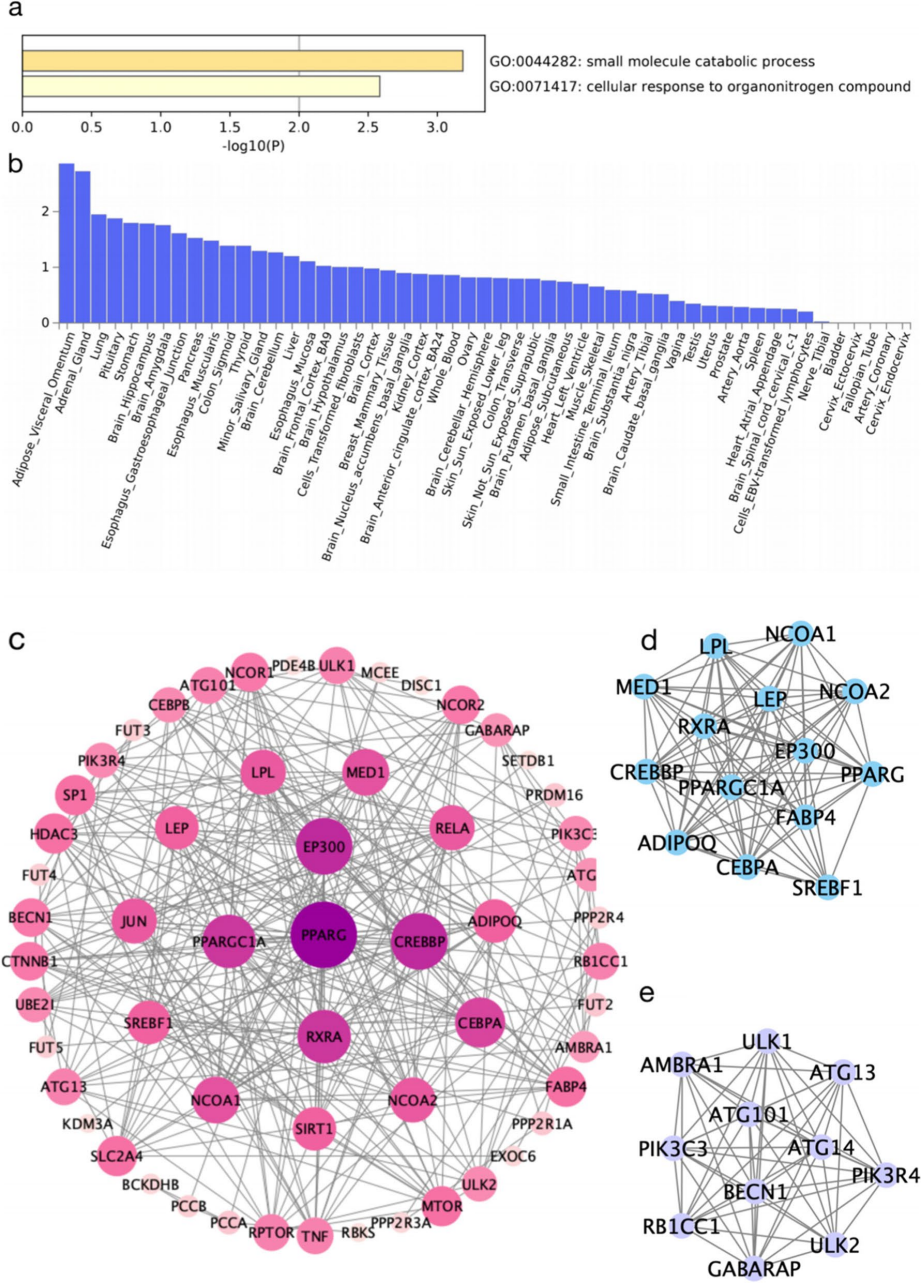
Functional enrichment analysis of shared genes between SCZ and TG. a Pathway enrichment analysis. b Tissue enrichment analysis using 53 tissues from the GTEx database (version 7). Significantly enriched differential expressed gene (DEG) sets (Bonferroni corrected *P* < 0.05) are highlighted in red. c PPI network for the shared genes between SCZ and TG. d and e Significant clusters related to the PPI network

Four pathways were significantly enriched in genes nearest identified genetic variants which were shared by SCZ and TG, including small molecule catabolic processes, cellular responses to organo-nitrogen compounds, cell responses to nitrogen compounds and inorganic cation transmembrane transport (Fig. 5a and Additional file 1 Supplementary Table 19).

GTEx enrichment analysis identified four independent tissues which were significantly enriched after Benjamini-Hochberg corrections for shared gene expression between SCZ and BMI: these included brain, liver, heart and kidney (Fig. 4b). The most strongly enriched tissue was the brain-anterior-cingulate-cortex. No significant tissue enrichment results were identified for shared genes between SCZ and TG (Fig. 5b).

### eQTL analysis of novel shared SNPs

Our eQTL analysis of novel shared SNPs was performed using the GTEx V7 database. *DERL2, SNX4, LY75* and *EFCAB6* expression levels were evaluated in brain, adipose and whole blood tissue in GTEx datasets. The risk allele rs13307-A was correlated with higher *LY75* expression levels in adipose, brain and whole blood tissue (Additional file 2 Supplementary Fig. 2 and Additional file 1 Supplementary Table 20). To identify the posterior probability of the *LY75* causal gene and phenotype at the same genetic variant rs13307, we performed tissue-eQTL colocalisation analyses and showed that eQTLs for *LY75* in the brain strongly colocalised with the genetic variant rs12469374, a perfect proxy of rs13307 (*R*^*2*^ = 0.90, *D′* = 0.98) in BMI (H4-Posterior Probability: 81.2%) and SCZ (H4-Posterior Probability: 82.1%) (Additional file 1 Supplementary Table 21).

Both rs1472584 and rs3130544 were not identified as *HCN1* and *C6orf15* eQTLs in brain, adipose, whole blood and liver tissue (Additional file 1 Supplementary Table 22).

### Protein-protein interaction (PPI) network analysis

To identify potential interactions between shared genes, the Search Tool for the Retrieval of Interacting Genes (STRING) database was used to perform PPI network analysis. The PPI network of genes shared between SCZ and BMI consisted of 219 nodes (genes) and 535 edges (interactions) (Fig. 4c). After using the Molecular Complex Detection (MCODE) plug-in to identify modules from the PPI network for shared genes between SCZ and BMI, the top central modules with MCODE scores > 10 were selected. Module 1, with scores of 12, consisted of 13 nodes and 72 edges (Fig. 4d). From Gene Ontology (GO) and Kyoto Encyclopaedia of Gene and Genomes (KEGG) pathway enrichment analysis, Module 1 was mainly enriched for cyclin-dependent protein serine/threonine kinase activity, transcription regulation of G1/S transition in the mitotic cell cycle, mitotic G1 DNA damage checkpoint signalling and cell cycle G1/S phase transition. PPI network analysis of genetic variants with concordant and opposite association directions identified one module (enriched for axon guidance, cellular responses to growth factors and cellular responses to oxidative stress) and one module (enriched for CNS neuron differentiation and positive regulation of peptidyl-tyrosine phosphorylation) for highly interconnected nodes, respectively (Additional file 2 Supplementary Fig. 3).

The PPI network for SCZ and TG shared genes consisted of 114 nodes (genes) and 699 edges (interactions) (Fig. 5c). After using the MCODE plug-in to identify modules from this PPI network, the top two central modules, with MCODE scores > 10, were selected. Module 1 had a score of 11.79, consisted of 30 nodes and 171 edges and was mainly enriched for genes involved in DNA-template transcription initiation, regulation of pri-miRNA transcription by RNA polymerase II, nuclear receptor activity, Huntington disease and RNA polymerase II-specific DNA-binding TF binding (Fig. 5d). Module 2, with a score of 10.4, consisted of 11 nodes and 52 edges, and was enriched for cell responses to fatty acids and adipocytokine signalling (Fig. 5e). PPI network analysis of concordant and opposite genes identified one module (enriched for cholesterol transport regulation) and two modules (module 1 was enriched for macroautophagy, the autophagosome, protein localisation to phagophore assembly sites and autophagosome assembly, and module 2 was enriched for the positive regulation of the mitotic cell cycle, top signalling and TOR signalling regulation), respectively (Additional file 2 Supplementary Fig. 4).

### Mendelian randomisation (MR) analysis

In MR analysis, when cardiometabolic traits were considered the exposure, no causal associations were observed between traits associated with SNPs for SCZ risk (Additional file 1 Supplementary Table 23). The test for horizontal pleiotropy, estimated using the MR-Egger intercept between BMI and SCZ, was significant (*P* = 0.049, Additional file 1 Supplementary Table 24) and indicated shared genetic variants between exposure and outcome. When cardiometabolic traits were considered an outcome, no causal relationship for SCZ was identified (Additional file 1 Supplementary Table 23).

## Discussion

In this study, we investigated the polygenic overlap between SCZ and two cardiometabolic traits (BMI and TG). The MR analysis indicated that genetic liability, the heritability of a disease, to SCZ exerted no influence on BMI|TG and vice versa, suggesting no evidence identified for a causal relationship between SCZ and BMI|TG. We identify 144 genetic variants between SCZ and BMI, and 15 genetic variants between SCZ and TG. Genetic variants were mainly enriched for neuronal system functions, including CNS neuron differentiation, BDNF signalling and the positive regulation of growth. Also, shared genetic variants demonstrated a mixture of concordant associations and associations with opposite directions between trait pairs. In total, 50% of shared SCZ SNPs had positive associations with BMI, while 46.7% had positive associations with TG. Additionally, we identified four novel genetic variants (rs3865350, rs9860913, rs13307 and rs9614186) shared by SCZ and BMI, and two novel genetic variants (rs1472584 and rs3130544) shared by SCZ and TG.

Shared genetic variants demonstrated a mixture of consistent and opposite association directions between trait pairs. This polygenic overlap between SCZ and BMI agreed with previous epidemiological association evidence [34, 35]. Also, 50% of genetic variants shared between BMI and SCZ had negative associations with BMI, and furthermore, 57% of genetic variants between BMI and SCZ were negatively associated with BMI when conjunctional FDR analysis was conducted at the 0.1 threshold (Additional file 1 Supplementary Table 25). These results agreed with genetic correlation analyses; we identified a negative correlation between SCZ and BMI (*rg* = − 0.081, *P* < 0.01, Additional file 1 Supplementary Table 26). Previous studies [5, 11] on SCZ and cardiometabolic traits indicated similar results with similar conclusions, however, in our study, we provided further evidence using different analytical approaches (HESS, partional LDSC and MR), thereby confirming associations may not be causal, but more likely pleiotropic in nature [11]. We also identified two chromosomal regions (chr11:27020461–28,481,593 and chr12:122007651–124,977,980; Fig. 2e and Additional file 1 Supplementary Table 2) with positive local genetic correlations and two genetic regions (chr16:29036613–31,382,943 and chr16:63691589–65,938,566; Fig. 2f and Additional file 1 Supplementary Table 3) with negative local genetic correlations, together with negative genetic correlations for DNase I hypersensitivity sites and TF binding sites (Additional file 1 Supplementary Table 27). MR analyses indicated no causal relationships between BMI and SCZ, but the test for horizontal pleiotropy, estimated using the MR-Egger intercept between BMI and SCZ, was significant (*P* = 0.049) and indicated shared genetic variants between the exposure (BMI) and outcome (SCZ). While underlying mechanisms remain unclear, one hypothesis suggests that poor nutrition, though subtle, may exert negative effects on neural development, leading to a increased incidence of mental health disorders, such as SCZ [11]. This observation suggested that variables such as antipsychotic treatments, dietary habits, or lifestyle may be primary factors contributing to weight gain in patients with long-term disease. Moreover, low BMI is viewed as a risk factor for SCZ [36], while a recent study reported an increased underweight frequency in patients with SCZ [37]. Our findings suggest variations in weight gain can occur during antipsychotic medication administration and may be partly mediated by genetics [38].

We performed several analyses, including eQTL, colocalisation, and functional annotation to identify a novel shared genetic overlap between SCZ and BMI|TG. Our eQTL analyses showed that the risk allele rs13307-A was associated with *LY75* expression in adipose and brain tissue. The genetic variant rs13307 for *LY75* in the brain strongly colocalised with BMI (H4-Posterior Probability: 81.2%) and SCZ (H4-Posterior Probability: 82.1%). Also, rs13307 may exert effects on the binding sites of TF motifs (Evi-1, RPEB-1 and RXR). This evidence suggested that the novel genetic variant rs13307 and the most proximate gene *LY75* exerted potential effects on SCZ and BMI comorbidity. A previous mouse study that used quantitative complementation, qualitative phenotypic and causal analysis, showed that an allele of the *LY75* locus potentially exerted pleiotropic effects on the weight of total and inguinal fat pads [39]. *LY75* is predominantly expressed by dendritic cells [40] and plays critical roles in endocytosis and T cell antigen presentation via major histocompatibility complex molecules, thereby contributing to immune function (e.g. antigen processing and complement pathways) [41, 42]. Recent studies suggested that chronic inflammation may be an important mediator linking metabolic abnormalities and severe mental illness [42, 43]. For example, elevated pro-inflammatory cytokines, including tumour necrosis factor-α and interleukin-6, were observed in patients with psychosis and CMD [43]. These findings support a potential role for *LY75*, which is involved in inflammation and immune pathways, in the shared genetic architecture of SCZ with cardiometabolic traits [44]. The exploration of potential mechanisms underlying combined TF motifs and *LY75* functions in SCZ and BMI is warranted.

We noticed that the minor allele frequency (MAF) of rs13307-A is 0.27 with the highest population frequency (1000 Genomes Phase 3, ESP and gnomAD) could be 0.49. The MAF of rs3865350-C is 0.26, with the highest population frequency (1000 Genomes Phase 3, ESP and gnomAD) could be 0.50. This indicates these alleles are more common in populations not merely in SCZ patients. In the QC process, numerous germline mutations were removed immediately after being generated, either by selection or randomly [45]. The retained variants may expand in the population; however, some mutations may cause disease and disorders owning to environmental changes over time, known as risk alleles [46]. Studies indicated that the deviation from 0.5 in the proportion of SNPs in which minor alleles were the risk alleles was relatively small (0.591–0.631) when the MAF was relatively high (> 0.1), indicating that most of the SNPs with those high MAFs were associated with diseases resulting from the changes of environment [47, 48]. Gorlov et al. suggested that environment or lifestyle-dependent diseases tend to have a higher frequency of risk-associated variants [49]. Besides, the analysis of the NHGRI-EBI Catalog data demonstrated that complex diseases, such as Alzheimer’s disease (late-onset), Parkinson’s disease, multiple sclerosis, metabolic syndrome, and schizophrenia, were more likely to have a high average risk allele frequencies [48]. In our study, the eQTL analyses indicated that the minor allele rs13307-A was associated with the expression of *LY75* in adipose and brain tissue, which may be a risk allele for the comorbidity of SCZ and BMI. However, since the rs13307-A with a high MAF, we speculate that recent environmental changes (including epigenetic changes or other factors) may play a crucial role, and these environmental changes should be considered for the study of the pathogenesis of comorbidity in future research.

Functional annotation of shared genetic variants showed that some genes were associated with gene expression in the brain and several biological and molecular processes, including CNS neuron differentiation, BDNF signalling, positive regulation of growth and the modulation of chemical synaptic transmission. A large proportion (approximately 67%) of shared genetic variants were brain-related and suggested that BMI regulation involved brain-related mechanisms [50]. Brain functions determine our behaviours as they determine lifestyle choices such as diet and exercise, which in turn affect BMI [51]. For example, BDNF signalling affects neural circuit structure and function and also modulates multiple neurotransmitter systems - functions closely related to SCZ [52]. Extensive evidence now suggests that *BDNF* is an essential contributor to food intake and body weight control [53]. Heterozygous *BDNF* knockout mice had reduced *BDNF* expression which led to age-dependent obesity and an insulin-resistant phenotype, and were characterised by elevated circulating levels of insulin, leptin and glucose [54, 55]. Critically, similar brain mechanisms may be involved in behaviours related to mental health disorders and BMI.

We failed to identify significant genetic correlations (*rg* = − 0.029, *P* = 0.187, Additional file 1 Supplementary Table 26) between SCZ and TG. However, from local genetic covariance analysis, two specific chromosomal regions were positively genetically correlated with SCZ and TG. These data were generally consistent with a previous meta-analysis on lipid profiles in first-episode psychosis patients, which reported higher TG levels [27]. In longitudinal studies [56, 57], dyslipidemia in patients with SCZ was typically studied as a side effect of antipsychotic medication. However, researchers reported that dyslipidemia and other metabolic risk factors may be present in early disease stages, before treatment initiation [58]. Thus, sustained elevated TG levels could mean dyslipidemia is associated with SCZ and not only due to medication [59]. Of note, a previous MR analysis supported the notion that SCZ was casually associated with increased TG levels [3], however, we did not identify a causal association between SCZ and TG.

Functional annotation analysis of shared genetic variants showed that variants contributed to both SCZ and TG, and were associated with small molecule catabolic processes, cell responses to organo-nitrogen compounds, cell responses to nitrogen compounds and inorganic cation transmembrane transport. A previous rat study reported that an inorganic cation transmembrane transport pathway was enriched in the cortex of rats treated with antipsychotic drugs [60]. In mice, cell responses to organo-nitrogen compounds and nitrogen compounds were involved in liver peroxisomal and mitochondrial roles to maintain TG balance and oxidative stress [61]. While we investigated pathways putatively involved in shared pathophysiology between SCZ and lipid traits, further experimental studies are warranted to elucidate the exact mechanisms.

Data from other phenotype pairs (TC|SCZ, HDL|SCZ, LDL|SCZ, T2D|SCZ, FG|SCZ and FIN|SCZ) (Additional file 4 Additional Tables and Additional file 5 Additional Figures) go beyond standard genetic association as shown by the findings in the conditional FDR and conjunctional FDR analyses that can evaluate the directions of association of the shared genetic variant. For example, despite the lack of genetic correlations for TC and SCZ (*rg* = − 0.0294), we identified 160 shared genetic variants (Additional file 3 Additional datasets). The shared genetic variant discovery could facilitate risk predictions for comorbid SCZ, thereby generating targeted interventions for cardiometabolic symptoms or similar diseases in patients with SCZ.

We failed to identify shared genetic variants between WHR, a measure of abdominal or visceral adiposity and SCZ, despite BMI being moderately correlated with WHR in adults (*rg* = 0.3) [62]. Some studies [12, 62] reported that SNPs related to body composition and fat distribution (WHR = waist and hip circumference) were also associated with mental disorders, and simultaneously associated with BMI, inconsistent with our study data. A possible explanation could be that our GWAS summary statistics for WHR were adjusted for BMI, and there is no or little isolated effect of body fat distribution on SCZ, as corroborated by several clinical and epidemiological studies [62–64].

Our study had some limitations. Firstly, our analyses were based on large-scale GWAS studies, which primarily consisted of Caucasian participants, thus in future work, we will extend our research remit to other ethnic groups. Secondly, in analyses using paired summary results, we could not control for other clinical factors, e.g., BMI when studying lipid traits. Thirdly, it was challenging to assess small effect sizes and speculate on molecular mechanisms underlying effective variants when examining potentially overlapping phenotypes. Overall, our methods enhanced the discovery of additional shared polygenic architectures and identified potential pleiotropic genes and biological pathways between two complex traits.

## Conclusions

We demonstrated a moderate to high genetic overlap between SCZ and BMI|TG, with a pattern of bidirectional associations, indicating a complex interplay of metabolism-related gene pathways in SCZ pathophysiology. Furthermore, we identified four novel SNPs and four most proximate genes which were shared by SCZ and BMI, and also two novel SNPs and two most proximate genes shared by SCZ and TG. Our findings contribute to a better understanding of the shared biological mechanisms underpinning SCZ and BMI|TG, and may facilitate reduced BMI and TG comorbidities among SCZ patients.

## Methods

### Study design, data summary and quality control (QC)

The overall study design is shown (Fig. 1). We retrieved summary statistics from publicly available GWAS studies, including SCZ [32] from the Psychiatric Genomics Consortium (*N* = 105,318 of European ancestry), BMI [65] (*N* = 236,231 of mixed ancestry) and WHR [66] (*N* = 348,501 of European ancestry) from the GIANT Consortium, T2D from the DIAGRAM Consortium [67] (*N* = 159,208 of European ancestry), fasting glucose (FG) [68] (*N* = 58,047 of European ancestry) and fasting insulin (FIN [68]) (*N* = 51,750 of European ancestry) from the MAGIC Consortium, and blood lipids (HDL [*N* = 99,900], LDL [*N* = 95,454 of European ancestry], TC [*N* = 100,184 of European ancestry] and TG [*N* = 96,598 of European ancestry]) [33] from the ENGAGE Consortium [69]. Dataset details are shown (Additional file 1 Supplementary Table 1).

We applied standardised GWAS summary data to minimise potential biases due to different array platforms and QC procedures. Firstly, we compared the md5 code of GWAS summary statistics and reported total SNPs to check data quality. Secondly, we used the LiftOver tool (http://genome.sph.umich.edu/wiki/LiftOver) to convert all GWAS summary data to the GRCh37/hg19 reference genome. Thirdly, we filtered out variants with a minor allele frequency (MAF) < 1% and removed SNPs with duplicates by keeping the first one. Lastly, we deleted SNPs with any missing values of OR/beta, SE, or *P*-values. Additionally, we restricted our analysis to autosomal chromosomes and European ancestry. The original number of SNPs and those remaining after QC are shown in Additional file 1 Supplementary Table 1.

### Pleiotropy analysis

Our pleiotropy analysis strategy was based on conditional FDR. Fold-enrichment plots are described elsewhere [70, 71]. Briefly, the conditional FDR method establishes an empirical Bayesian statistical framework and uses GWAS summary statistics from the trait of interest (e.g. SCZ) together with statistics for a conditional trait (e.g. BMI), to estimate the posterior probability that an SNP has no association with the primary trait, given that the *P*-value for that SNP in both primary and conditional traits are as small as, or smaller than the observed *P*-value. Fold-enrichment plots graphically depicted pleiotropy by showing fold-enrichment in terms of SNP numbers on the ordinate, and nominal -log10(P) values for associations with SCZ on the abscissa [7]. Separate curves were established for SNP subsets which reached specific significance levels for associations with SCZ.

Our analyses followed two directions; firstly, with SCZ as the primary phenotype A and cardiometabolic traits as the conditional phenotype B, then vice versa for the second direction. We also generated a fold-enrichment plot to assess polygenic overlap between SCZ and cardiometabolic risk traits.

### Local genetic covariance analysis

To investigate if a local genetic correlation existed between SCZ and cardiometabolic traits, the HESS [72] package was used to estimate local genetic correlations between a pair of traits at each LD-independent region in the genome. Approximate independent LD blocks, averaging 1.5 Mb in length, were used to calculate each local genetic heritability trait and genetic covariance. A total of 1685 approximate LD-independent genomic regions (excluding the major histocompatibility complex region) were used for analysis. Genomic regions were also excluded if the estimated local single-trait heritability was negative due to insufficient study power.

### Conjunctional FDR analysis

To determine if genetic variants were likely to be shared by two phenotypes, we computed conjunctional FDR statistics. The conjunctional FDR is an extension of the conditional FDR and is defined as the maximum of two conditional FDR statistics for a specific SNP. The conjunctional FDR estimates the posterior probability that an SNP is null for either trait or both, given that the *P*-values for both phenotypes are as small as, or smaller than the *P*-values for each trait individually. More details are provided elsewhere [73–75]. In our study, we included shared SNPs with conjunctional FDR < 0.05. Manhattan plots were constructed based on the conjunctional FDR to show the genomic location of shared genetic variants. Traits were selected based on two criteria: 1) genetic pleiotropy existed in two phenotypes and 2) local genetic covariance analysis was significant in at least one region. Two trait pairs met these criteria: SCZ vs. BMI and SCZ vs. TG.

### Functional annotations

We mapped SNPs identified by conjunctional FDR to promising genes using ANOVAR software [76] and described the distribution of shared SNPs. We extracted novel shared SNPs with strong LD (*r*^*2*^ ≥ 0.8) with the index variant based on the 1000 Genomes Phase 1 European individuals from the online HaploReg v4.2 tool [77]. Using data from ENCODE [78] and Roadmap [79] databases, we predicted regulatory elements (promoters and enhancers, etc.) using histone modification markers (H3K4me3, H3K4me1 and H3K27ac), chromatin state segmentation and DNase I hypersensitivity sites (DHS) in 125 cell types.

Functional Mapping and Annotation of GWAS (FUMA https://fuma.ctglab.nl/) was used to annotate significantly shared lead SNPs with functional categories using combined annotation dependent depletion scores (CADD) [80], RegulomeDB scores and chromatin states. A CADD score > 12.37 indicated a deleterious protein association with outcomes. The RegulomeDB score indicated the regulatory functionality of SNPs, based on the expression of quantitative trait loci (eQTL) and chromatin markers. The chromatin state indicated the accessibility of genomic regions using 15 categories as predicted by ChromHMM and based on five chromatin markers for 127 epigenomes. GTEx tissue enrichment analysis was based on 53 general tissue types and conducted using FUMA [81]. The Genetic Variants Allelic TF Binding Database (GVATdb) was used to search for SNPs with differential TF binding (http://renlab.sdsc.edu/GVATdb/search.html) capabilities and was also used to characterise the allelic binding of common human SNPs (MAF > 1% in European and Asian populations) to distinct TFs. The transcriptional regulation in the context of enhancers (EnhancerDB) resource was used to define tissue-specific enhancers by setting threshold scores for tissue-specific enhancers (http://lcbb.swjtu.edu.cn/EnhancerDB/).

### Bioinformatics analysis

To understand the biological role of genes nearest the shared genetic variants between SCZ and cardiometabolic traits, we performed multiple post-GWAS functional analyses in shared genes identified by conjunctional FDR. We used the Metascape tool [82] (http://metascape.org), with default parameters, to assess the overrepresented enrichment of shared gene sets between SCZ and cardiometabolic traits (BMI and TG) in KEGG pathway analyses (www.kegg.jp/kegg/kegg1.html), GO Biological Processes, GO Cellular Components, GO Molecular Functions, WikiPathways, Hallmark and Reactome gene sets.

To evaluate the possible effects of genetic variants on transcriptional activity, we performed an eQTL analysis using the GTEx V7 database [83]. Previous studies indicated that SCZ and BMI-associated genetic variants showed strong gene expression enrichment in brain tissues [84, 85]. As for other cardiometabolic traits, including obesity and blood lipids, these were reportedly stored in subcutaneous adipose tissue [84]. The liver is related to fat metabolism and secretes TG [86]. Considering that genetic variants may affect gene expression in a tissue-specific manner, eQTL analyses were performed on brain, adipose and whole blood tissue to identify shared SNPs between SCZ and BMI, while the GTEx database was used for the same tissue and liver to identify shared SNPs between SCZ and TG.

### Colocalisation between GWAS and eQTL signals

Colocalisation analysis was performed to test the probability of genetic variant the same one of GWAS and tissue-specific eQTL using GTEx dataset. Summary statistics for SNPs (regardless of GWAS *P*-value) within 200 kb of significant lead SNPs and common to both GWAS and eQTL studies, were inputted into coloc under default parameter settings [87]. This approach tested the probability of five hypotheses (H0–4), of which H_4_ tested the hypothesis that the same causal variant was shared between GWAS and tissue-specific eQTLs. Genetic variants with 80% or higher probability for H_4_ were compared to understand the LD structure and the most prominent variant being shared by GWAS and eQTL [88].

### Protein-protein interaction (PPI) networks

PPI networks of shared genes were generated using the STRING 11.0; https://string.embl.de/) database, with a confidence score = 0.7 (high confidence) [89]. The PPI network was visualised using Cytoscape 3.8.2 software, and network modules were screened using the MCODE plug-in with MCODE scores > 10 [90]. The connectivity cut-off degree = 2, node score cut-off = 0.2, k-core = 2 and a maximum depth of 100 was permitted [91]. Pathway enrichment analysis of genes in modules was performed and *P* < 0.05 was considered a statistically significant difference.

### MR analysis

To test for causal relationships between SNPs identified by conditional analysis, we performed MR analysis [92] using cardiometabolic trait GWAS SNPs as instrumental variables in the ‘TwoSampleMR’ package. For the exposure, default parameter settings of a *P* threshold of 5 × 10^− 8^, LD *R*^*2*^ = 0.001 and clumping distance = 10 kb were used [93]. We conducted MR using five approaches: inverse variance weighted (IVW), MR-Egger, weighted median, simple mode and weighted mode methods.

In the main analysis, we reported IVW estimates, however, if Egger’s method identified horizontal pleiotropy (e.g., SNPs associate with exposure but influence the outcome through pathways not specific to exposure), then MR-Egger results were used.

## Supplementary Information

**Additional file 1.** **Supplementary Tables.**

**Additional file 2.** **Supplementary Figures.**

**Additional file 3.** **Additional datasets.**

**Additional file 4.** **Additional Tables.**

**Additional file 5.** **Additional Figures.**

**Additional file 6.** **Supplementary Methods.**

**Additional file 7.** **Supplementary Results.**

## Data Availability

The datasets analysed during the crrent study are available in the following public domain resources: https://www.ebi.ac.uk/gwas/publications/25673413 (Accession Number: GCST002783); https://www.ebi.ac.uk/gwas/publications/31453325 (Accession Number: GCST009127); https://www.ebi.ac.uk/gwas/publications/20081858 (Accession Number: GCST000571 and GCST000568); https://www.ebi.ac.uk/gwas/publications/20686565 (Accession Number: GCST000758, GCST000760, GCST000759, and GCST000755); https://figshare.com/articles/dataset/scz2018clozuk/14681220 (Data DOI: 10.6084/m9.figshare.14681220).
