# Supplementary material for: Exploring Lead loci shared between schizophrenia and Cardiometabolic traits
Source: BMC Genomics. 2022 Aug 25;23:617. doi: 10.1186/s12864-022-08766-4 (PMC9414090; doi:10.1186/s12864-022-08766-4)
Supplement: Supplementary file 2 — Additional file 2. Supplementary Figures. [file 12864_2022_8766_MOESM2_ESM.docx]

**Supplementary Figures**


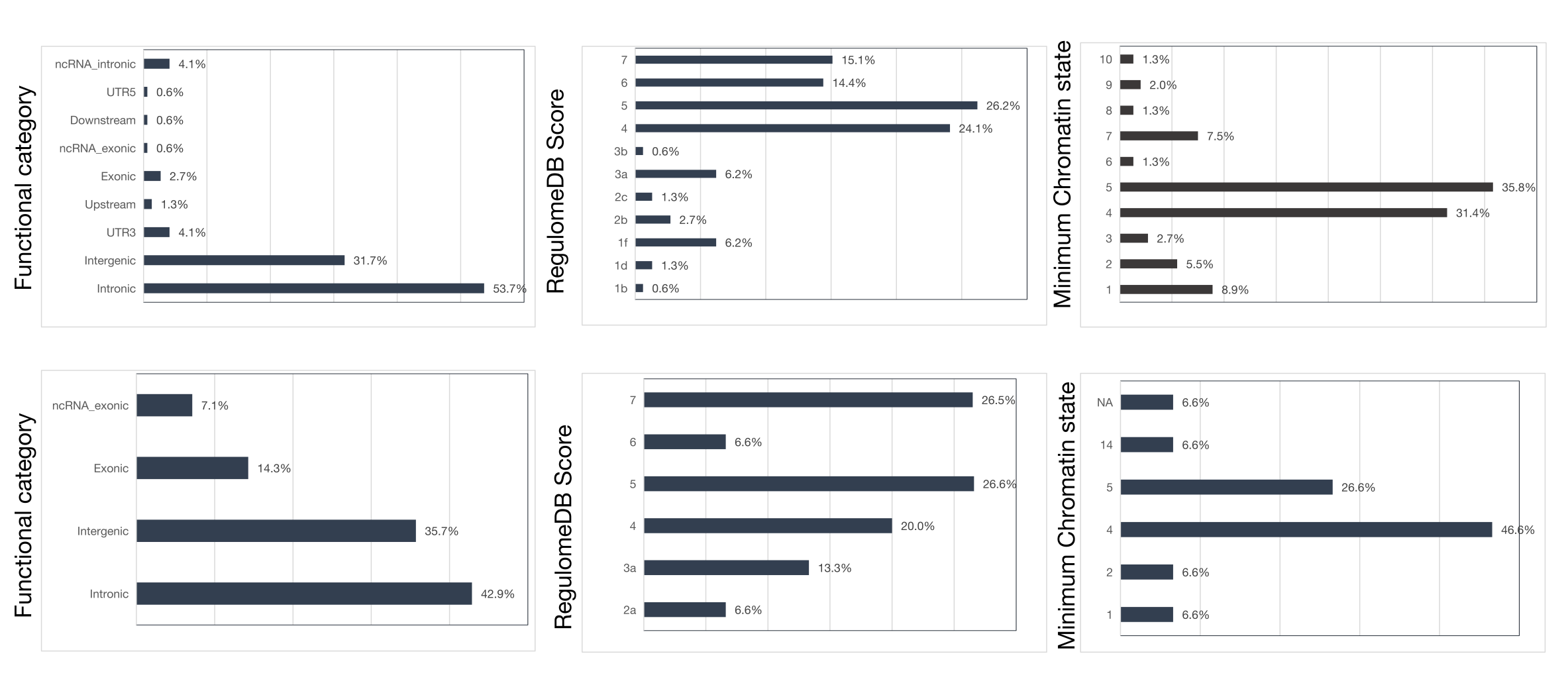


b. TG and SCZ

a. BMI and SCZ

Supplementary Figure 1. Distribution of the annotation for lead SNPs shared between BMI and TG and SCZ at conjunctional false discovery rate less than 0.05


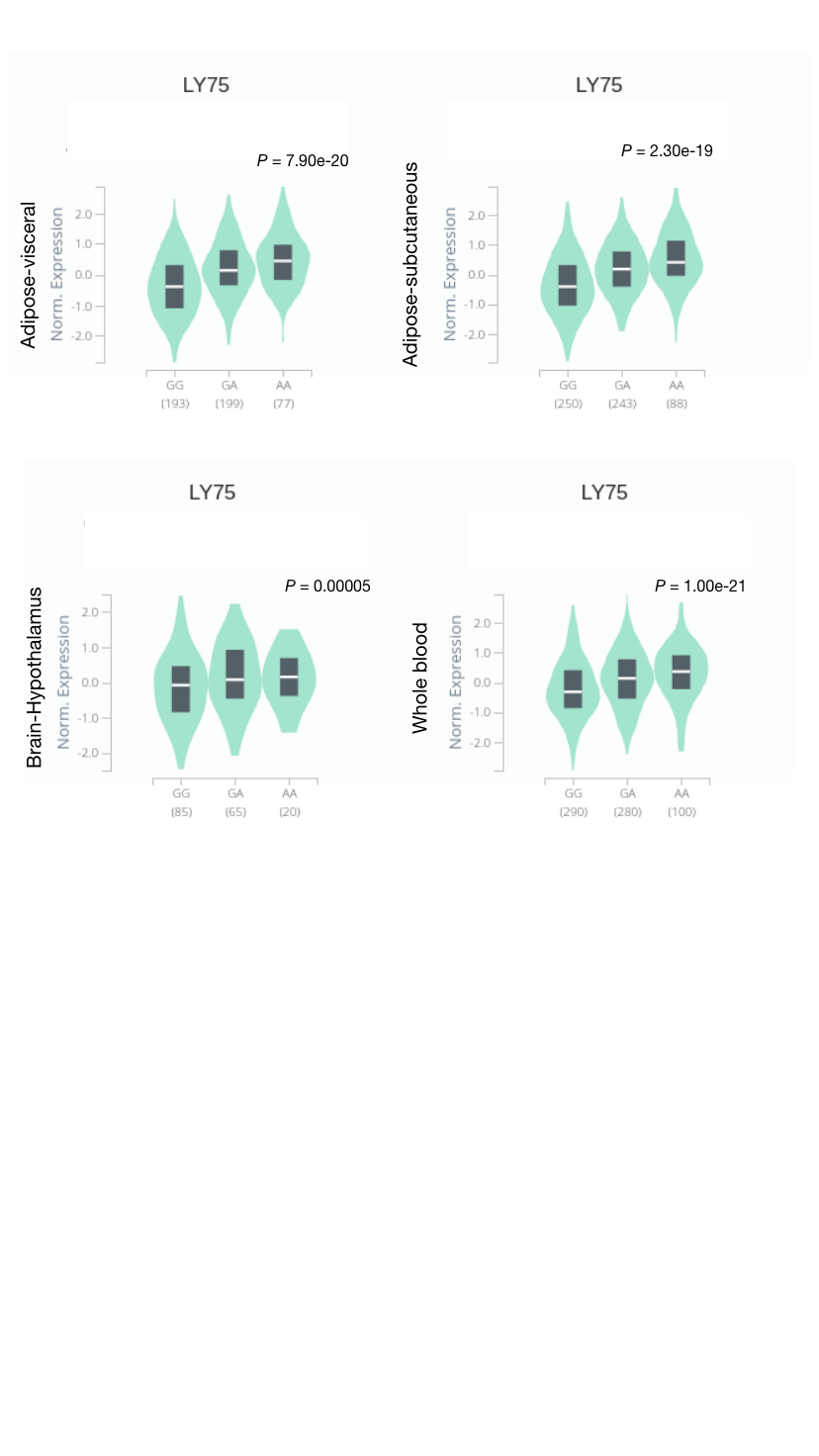


*LY75*

*LY75*

*LY75*

*LY75*

Supplementary Figure 2. Violin plots for eQTL analysis results in adipose, brain and whole blood for novel shared SNP (rs13307) between SCZ and BMI.


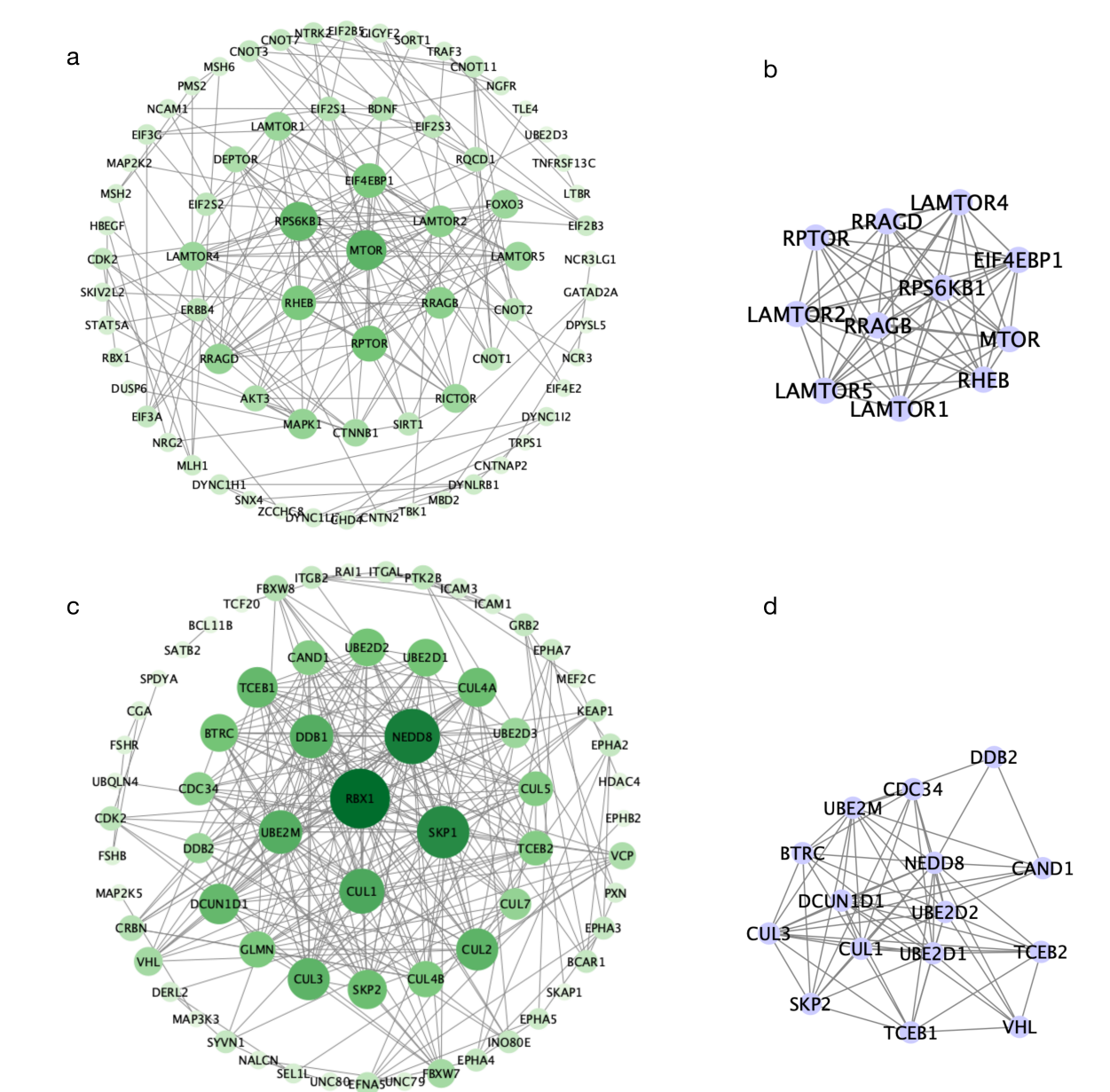


Supplementary Figure 3. The PPI network of concordant and opposite genes of SCZ and BMI. (a) The PPI network for the concordant genes shared by SCZ and BMI. (b) Significant cluster related to the concordant PPI network. (c) The PPI network for the opposite shared genes by SCZ and BMI. (d) Significant cluster related to the opposite PPI network.


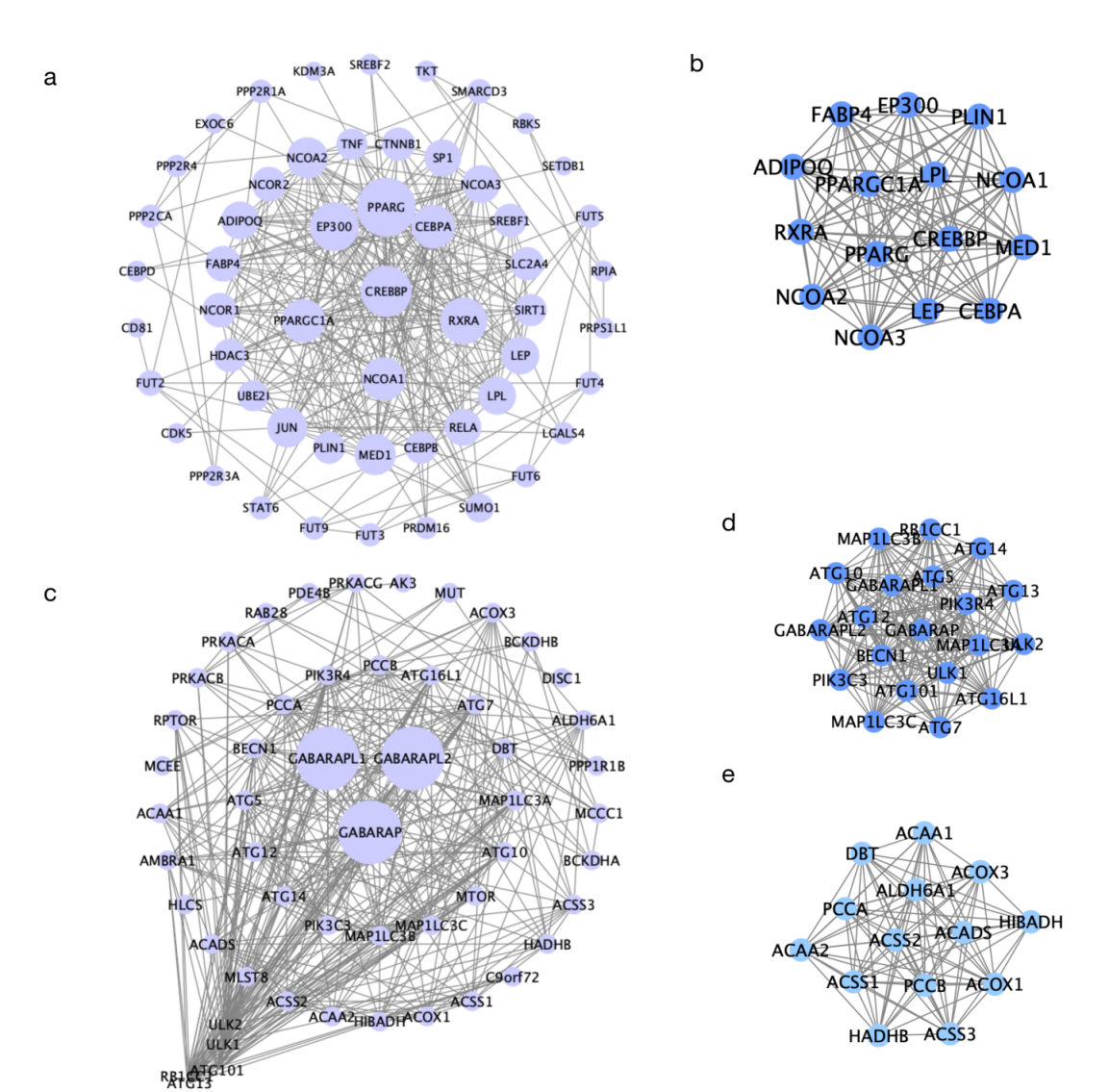


Supplementary Figure 4. The PPI network of concordant and opposite genes of SCZ and TG. (a) The PPI network for the concordant genes shared by SCZ and TG. (b) Significant cluster related to the concordant PPI network. (c) The PPI network for the opposite shared genes by SCZ and TG. (d)(e) Significant clusters (1,2) related to the opposite PPI network.
