# Supplementary material for: Exploring Lead loci shared between schizophrenia and Cardiometabolic traits
Source: BMC Genomics. 2022 Aug 25;23:617. doi: 10.1186/s12864-022-08766-4 (PMC9414090; doi:10.1186/s12864-022-08766-4)
Supplement: Supplementary file 5 — Additional file 5. Additional Figures. [file 12864_2022_8766_MOESM5_ESM.docx]

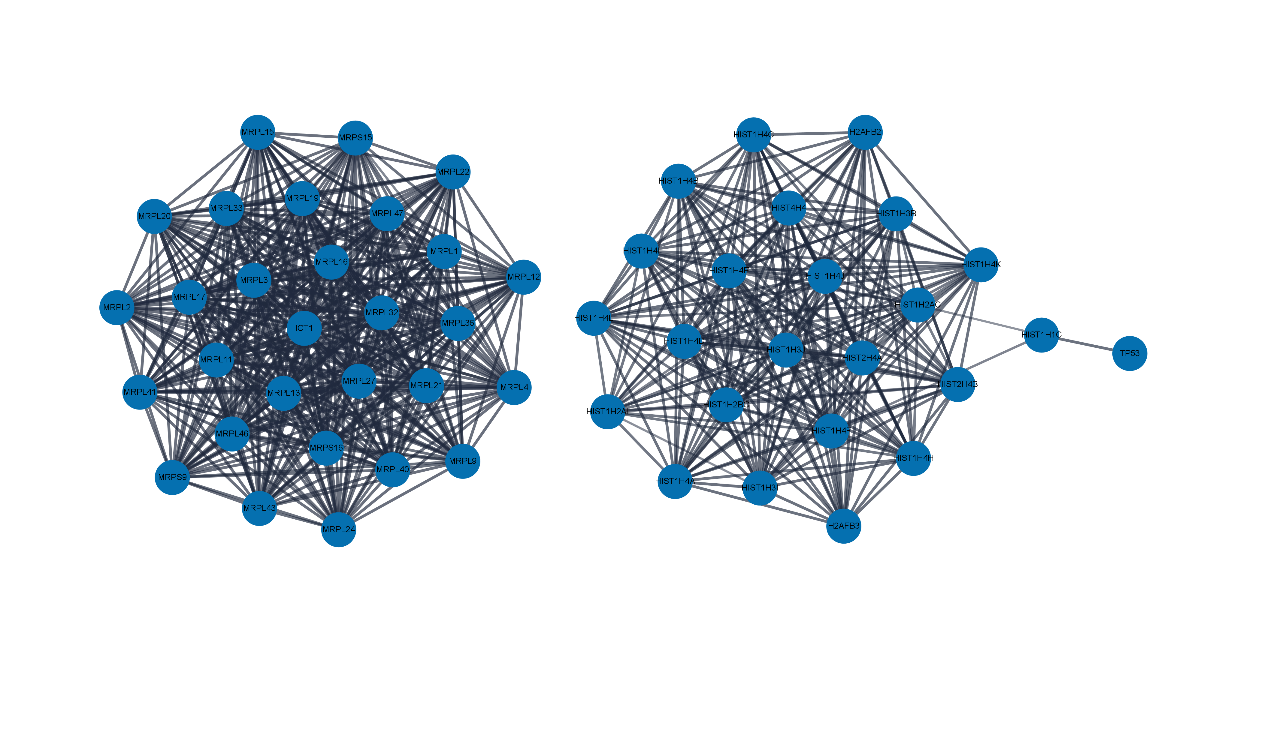

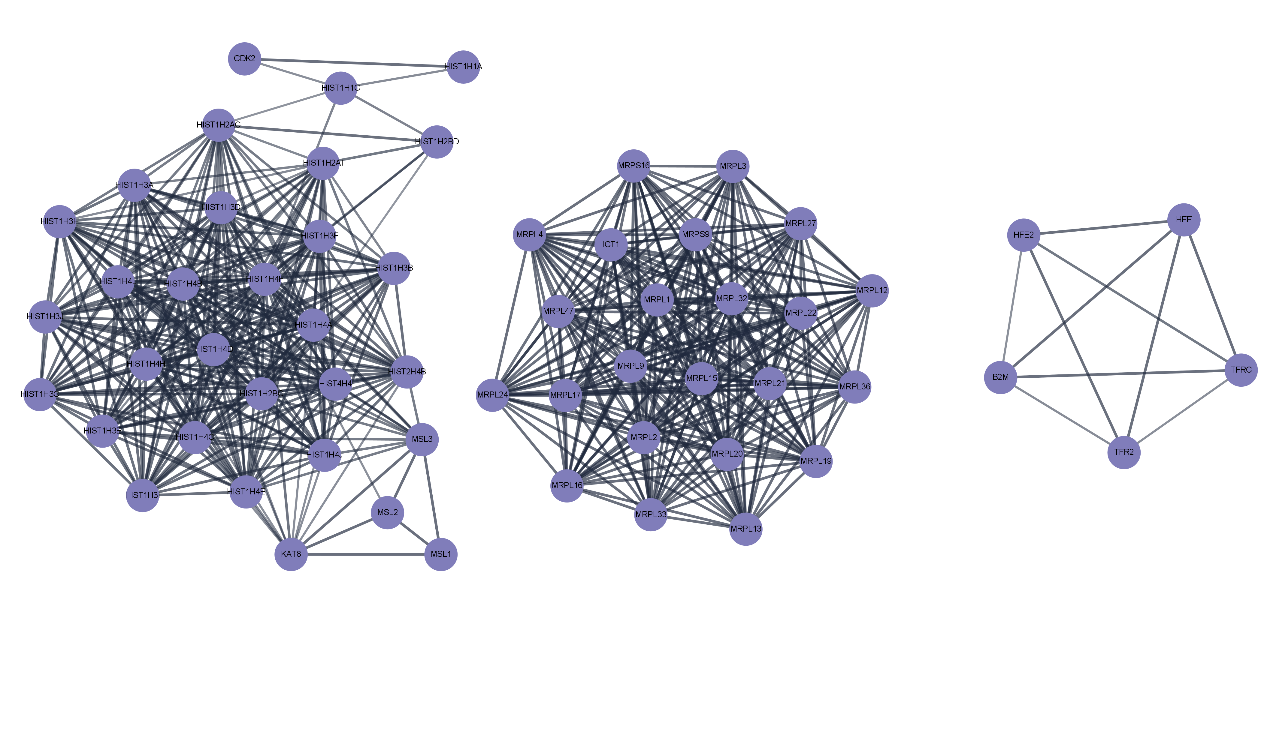


bb

a

Additional Figure 1. The PPI network of concordant and opposite genes of SCZ and TG. (a) The PPI network for the concordant genes shared by SCZ and TG. (b) The PPI network for the opposite shared genes by SCZ and TG.


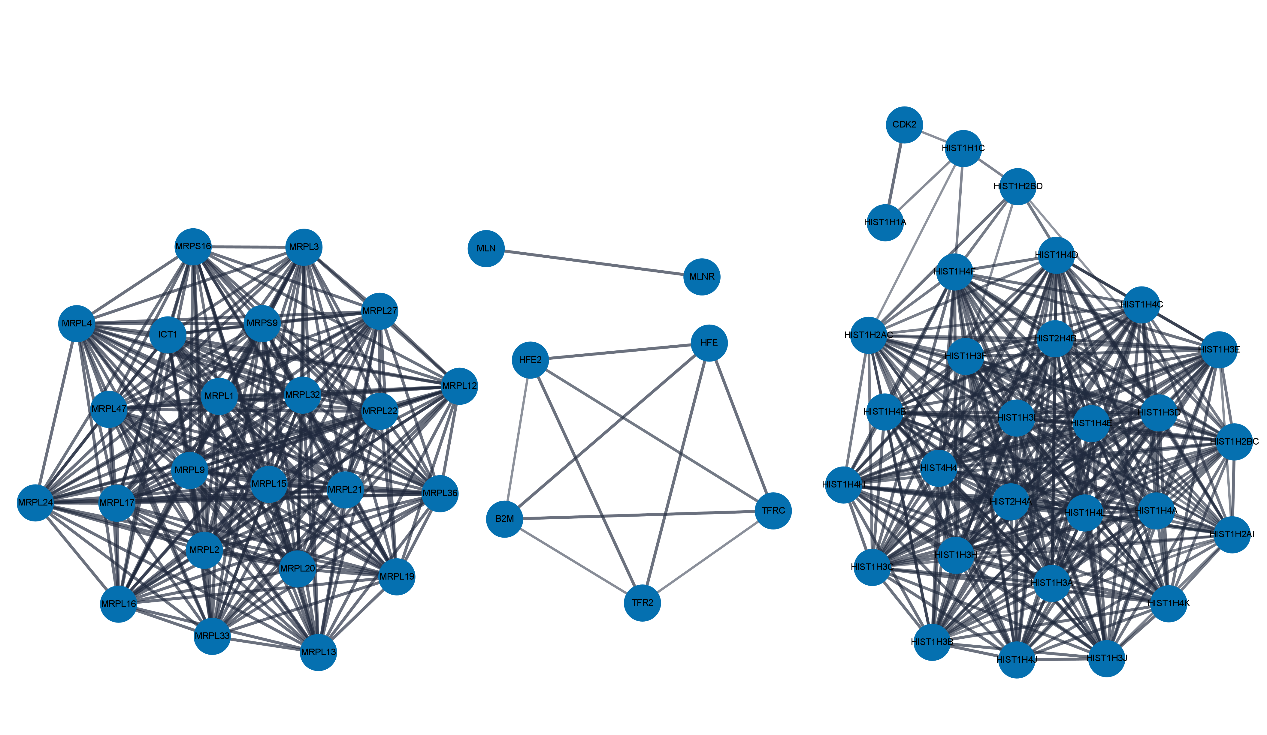

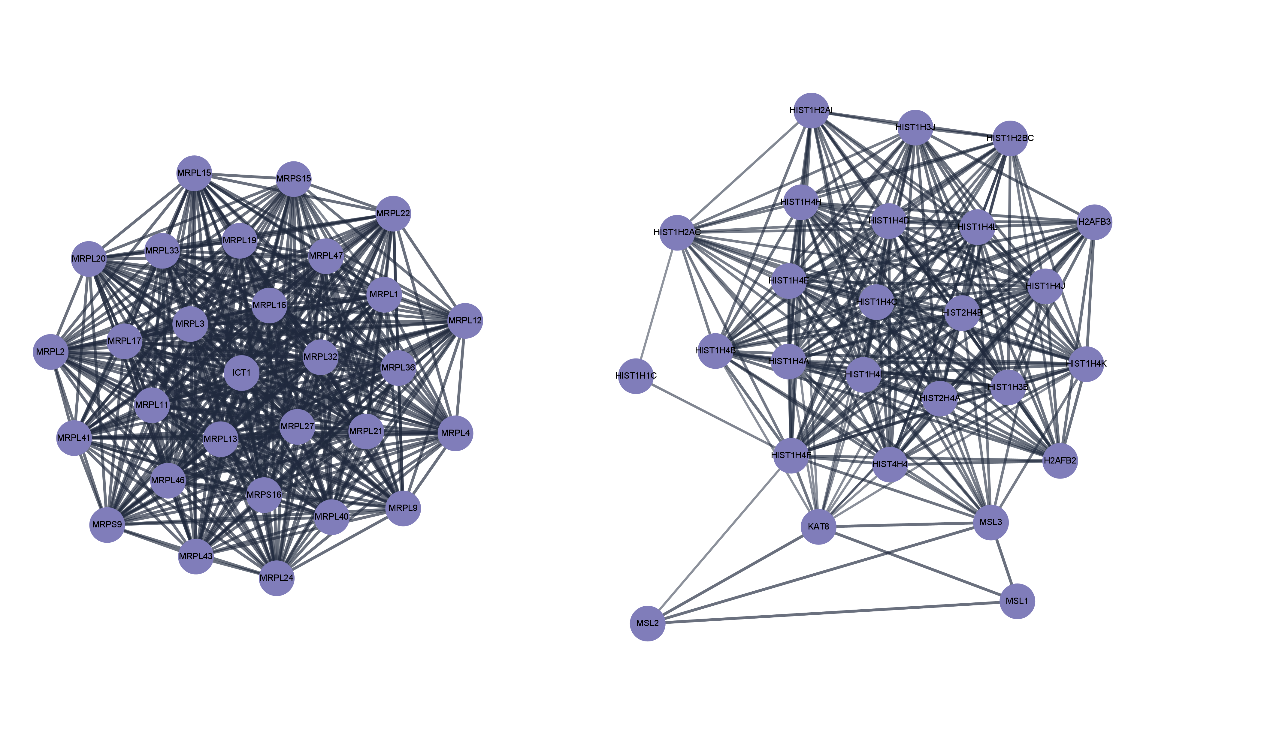


b

a

Additional Figure 2. The PPI network of concordant and opposite genes of SCZ and HDL. (a) The PPI network for the concordant genes shared by SCZ and HDL. (b) The PPI network for the opposite shared genes by SCZ and HDL.
