# Supplementary material for: Exploring Lead loci shared between schizophrenia and Cardiometabolic traits
Source: BMC Genomics. 2022 Aug 25;23:617. doi: 10.1186/s12864-022-08766-4 (PMC9414090; doi:10.1186/s12864-022-08766-4)
Supplement: Supplementary file 6 — Additional file 6. Supplementary Methods. [file 12864_2022_8766_MOESM6_ESM.docx]

**Supplementary methods**

***LD score regression analysis***

We conducted post-GWAS genome-wide genetic correlation analysis by LD score regression (LDSC) using all SNPs after merging with HapMap3 SNP excluding the HLA region. LDSC estimates genetic correlation between the true causal effects of two traits (ranging from −1 to 1) from summary statistics using the fact that the GWAS effect size estimate for each SNP represents the effects of all SNPs in linkage disequilibrium with that SNP. SNPs in a high linkage disequilibrium region would have higher χ2 statistics than SNPs in a low linkage disequilibrium region, and a similar relationship is observed when single-study test statistics are replaced with the product of the z-scores from two studies of traits with some correlation[1]. LDSC applied a self-estimated intercept during the analysis to account for shared subjects between studies [2].

***Partitioned genetic correlation analysis***

To characterize the genetic overlap at the level of functional categories, we estimated genetic correlation between SCZ and BMI in 9 large genomic functional annotation using partitioned LDSC, where each annotation contains more than 200,000 SNPs that are in common with our GWAS data. These annotations included transcribed region, transcription factor binding sites (TFBS), Super Enhancer, intron, DNaseI digital genomic footprinting (DGF) region, DNase I hypersensitivity sites (DHSs) and histone marks H3K9ac, H3K4me1, H3K4me3, H3K27ac [3]. For each annotation, we re-calculated LD scores for SNPs assigned to that particular category and then used the annotation-specific LD scores for estimating the SCZ|BMI trait genetic correlation for each partition separately.

***Functional annotation***

We functionally annotated all candidate SNPs in the genomic loci with a condFDR or conjFDR value <0.1 and an LD r2≥0.6 with any of the independently associated SNPs using FUMA. We annotated SNPs using Combined Annotation Dependent Depletion (CADD) scores8, RegulomeDB scores9, and chromatin states[4, 5]. The CADD score predicts the deleteriousness of SNPs based on 63 functional annotations.[6] A CADD score of 12.37 is the threshold for being considered deleterious. The RegulomeDB score shows the regulatory functionality of SNPs based on eQTLs and chromatin marks. The chromatin state characterizes a genomic region’s accessibility with 15 categorical states predicted by ChromHMM based on 5 chromatin marks for 127 epigenomes.

The Genotype-Tissue Expression (GTEx V7) project[7] was established to characterize human transcriptomes and has created a reference resource of gene expression levels from non-diseased tissues, including genotype, gene expression, and histological data for 449 human donors across 53 tissue types[8]. Pre-calculated differentially expressed genes (DEG) sets were created for each of expression data set. DEG sets are defined by a two-side t tests per label versus all remaining (tissue types or developmental stages). Genes with a Bonferroni corrected p-value < 0.05 and absolute log fold change > 0.58 are selected as DEG. The -log10 (P values) in the graph refer to the probability of the hypergeometic test.

***Genomics bioinformatics analysis***

In order to understand the biological insights of the genes nearest the identified shared loci between SCZ and cardiometabolic traits, we performed multiple post-GWAS functional analyses among shared genes identified by the conjunctional FDR. We used the Metascape tool[9] (http://metascape.org), with default parameters, to assess overrepresented enrichment of the identified shared gene set between SCZ and cardiometabolic traits (BMI and TG) in the KEGG Pathway, Gene Ontology (GO) Biological Processes (BP), GO Cellular Component (CC), GO Molecular Function (MF), WikiPathways, Hallmark Gene sets, and Reactome Gene Sets. All genes in the genome were used as the enrichment background. Using default parameters of the Metascape tool, terms with *P* < 0.01, minimum count 3, and enrichment factor > 1.5 (the enrichment factor being the ratio between observed count and the count expected by chance) were collected and grouped into clusters based on their membership similarities. More specifically, *P* values were calculated based on accumulative hypergeometric distribution; q values were calculated using the Benjamini-Hochberg procedure to account for multiple testing.

**References**

1. Bulik-Sullivan B, Finucane HK, Anttila V, et al. Genetic Consortium for Anorexia Nervosa of the Wellcome Trust Case Control C, Duncan L, Perry JR, Patterson N, Robinson EB, Daly MJ, Price AL, Neale BM (2015a) An atlas of genetic correlations across human diseases and traits. Nat Genet 47: 1236–41. doi: 10.1038/ng.3406
2. Bulik-Sullivan BK, Loh PR, Finucane HK, Ripke S, Yang J, Schizophrenia Working Group of the Psychiatric Genomics C, Patterson N, Daly MJ, Price AL, Neale BM (2015b) LD Score regression distinguishes confounding from polygenicity in genome-wide association studies. Nat Genet 47: 291–5. doi: 10.1038/ng.3211
3. Finucane HK, Bulik-Sullivan B, Gusev A, et al. Schizophrenia Working Group of the Psychiatric Genomics C, Consortium R, Purcell S, Stahl E, Lindstrom S, Perry JR, Okada Y, Raychaudhuri S, Daly MJ, Patterson N, Neale BM, Price AL (2015) Partitioning heritability by functional annotation using genome-wide association summary statistics. Nat Genet 47: 1228–35. doi: 10.1038/ng.3404
4. Correll CU, Robinson DG, Schooler NR, Brunette MF, Mueser KT, Rosenheck RA, et al. Cardiometabolic Risk in Patients With First-Episode Schizophrenia Spectrum Disorders: Baseline Results From the RAISE-ETP Study. Jama Psychiat. 2014;71:1350–63.
5. Andreassen OA, Djurovic S, Thompson WK, Schork AJ, Kendler KS, O’Donovan MC, et al. Improved Detection of Common Variants Associated with Schizophrenia by Leveraging Pleiotropy with Cardiovascular-Disease Risk Factors. Am J Hum Genetics. 2013;92:197–209.
6. Bahrami S, Steen NE, Shadrin A, et al. Shared Genetic Loci Between Body Mass Index and Major Psychiatric Disorders: A Genome-wide Association Study. JAMA Psychiatry. 2020;77(5):503–512. doi:10.1001/jamapsychiatry.2019.4188.
7. Aguet F, Brown AA, Castel SE, Davis JR, He Y, Jo B, et al. Genetic effects on gene expression across human tissues. Nature. 2017;550:204–13.
8. Watanabe K, Taskesen E, Bochoven A van, Posthuma D. Functional mapping and annotation of genetic associations with FUMA. Nat Commun. 2017;8:1826.
9. Zhou Y, Zhou B, Pache L, Chang M, Khodabakhshi AH, Tanaseichuk O, et al. Metascape provides a biologist-oriented resource for the analysis of systems-level datasets. Nat Commun. 2019;10:1523.
