# Supplementary material for: Exploring Lead loci shared between schizophrenia and Cardiometabolic traits
Source: BMC Genomics. 2022 Aug 25;23:617. doi: 10.1186/s12864-022-08766-4 (PMC9414090; doi:10.1186/s12864-022-08766-4)
Supplement: Supplementary file 7 — Additional file 7. Supplementary Results. [file 12864_2022_8766_MOESM7_ESM.docx]

**Supplemetary Results**

**Genetic variants and genes identified by conjunctional false discovery rate analysis shared between SCZ and cardiometabolic traits**

To identify genetic variants shared between SCZ and TC, HDL, LDL, FG, FIN, T2D and WHR, we performed conjunctional FDR analysis. The conjunctional FDR is an extension of the conditional FDR and is defined as the maximum of the two conditional FDR statistics for a specific SNP. The conjunctional FDR estimates the posterior probability that a SNP is null for either trait or both, given that the *P* values for both phenotypes are as small as or smaller than the *P* values for each trait individually. A total of 160 distinct genetic variants were shared between SCZ and TC at conjunctional FDR < 0.05 (Additional datasets), mapping the most proximate 29 genes. All of these genes have been previously reported to be associated with TC or SCZ. A total of 149 distinct genetic variants were shared between SCZ and HDL at conjunctional FDR < 0.05 (Additional datasets), mapping the most proximate 28 genes. All of these genes have been previously reported to be associated with HDL or SCZ. A total of 35 distinct genetic variants were shared between SCZ and LDL at conjunctional FDR < 0.05 (Additional datasets), mapping the most proximate 6 genes. All of these genes have been previously reported to be associated with LDL or SCZ. A total of 30 distinct genetic variants were shared between FG and SCZ at conjunctional FDR < 0.05 (Additional datasets), mapping the most proximate 3 genes. All of these genes have been previously reported to be associated with FG or SCZ. We only identified 1 genetic variant shared between WHR or T2D and SCZ, mapping the most 1 gene. We failed to identied shared genetic variants between FIN and SCZ at onjunctional FDR < 0.05 (Additional datasets).

**Annotation of Genetic Variants Shared Between SCZ and cardiometabolic traits**

The functional annotation of all SNPs at conjunctional FDR < 0.05 for SCZ and cardiometabolic traits are shown in Supplement1. Most of the SNPs are within intronic (73.7%) and intergenic (11.1%) regions, and 13.6% had a RegulomeDB score < 3, predicting potential regulatory functions (Additional datasets).

**Pathway Analysis of Genetic Variants Shared Between SCZ and cardiometabolic traits**

To determine the overrepresented pathways among the genes nearest the identified genetic variants shared between SCZ and cardiometabolic traits, we carried out pathway overrepresentation analyses for shared genetic variants between SCZ and cardiometabolic traits. We only got significant overpresented pathways in TC|SCZ (Additional Tables Table A1,2) and HDL|SCZ (Additional Tables Table A3,4) phenotype pairs. 49 pathways were significantly overrepresented among the genes nearest the identified genetic variant shared between SCZ and TC. The top 3 enriched pathways were related with sialic acid transport, nucleosome assembly and chromatin assembly (Additional Tables Table A5). The analyses of genes with concordant and opposite association directions seperately between TC and SCZ indicate 60% overlap in the overrepresented pathways. The concordant genes were enriched in the sialic acid transmembrane transporter activity, sialic acid transport and sodium:phosphate symporter activity in concordant and opposite associations, separately (Additional Tables Table A6,7).

10 pathways were significantly overrepresented among the genes nearest the identified genetic variant shared between SCZ and HDL. The top 3 enriched pathways were related with sialic acid transmembrane transporter activity, sialic acid transport and sodium:phosphate symporter activity (Additional Tables Table A8). The analyses of genes with concordant and opposite association directions separately between HDL and SCZ indicated only great overlap in the overrepresented pathways. The concordant and opposite genes were enriched in sialic acid transmembrane transporter activity, sialic acid transport, and solute:cation symporter activity (Additional Tables Table A9).

**PPI network analysis**

To seek the potential interactions between shared genes, the STRING tool was employed to perform PPI network analysis. The PPI network of genes shared between SCZ and TC consisted of 79 nodes (genes) and 125 edges (interactions). After using MCODE plug-in to identify modules from the PPI network for the concorant shared genes between SCZ and TC, the top central module with MCODE scores > 10 were selected. Module 1 with scores of 17 consisted of 16 nodes and 72 edges (Additional Figures Additional Figure1a). PPI network analysis of genetic variants with concordant and opposite association directions identified 1 module ith scores of 13 consisted of 13 nodes and 23 edges (Additional Figures Additional Figure1b).

The PPI network of genes shared between SCZ and consisted of 102 nodes (genes) and 249 edges (interactions). After using the MCODE plug-in to identify modules from the PPI network for the concorant shared genes between SCZ and HDL, and the top two central modules with MCODE scores > 10 were selected. Module 1 had a score of 10.2 and consisted of 14 nodes and 65 edges. Module 2, with score of 18, consisted of 20 nodes and 98 edges (Additional Figures Additional Figure2a). PPI network analysis of opposite genes identified 1 module (Additional Figures Additional Figure2b).
